# Abstract of the Proceedings of the Buffalo Medical Association

**Published:** 1858-08

**Authors:** Austin Flint


ART. VIII.—*Abstract of Proceedings of the Buffalo Medical Association.*

TUESDAY EVENING, July 6, 1858.

The Association met.

Present—The President, Dr. Wyckoff, in the chair; Drs. Newman, Flint, Mixer, Nichell, Eastman, Hamilton, Treat, Whitehead, Wilcox, Rochester, White, Rankin, Monahan, and Flint, Jr.

The minutes of the last meeting were read and approved.

*Prof. Rochester,* chairman of the committee on "the presentation of medical accounts," read the following report:

The committee appointed to report on "the presentation of medical accounts," respectfully submit the following statement:

It has been, and still is, the general but not the invariable usage of the profession in this country, to render their accounts annually or semi-annually only. This custom originated mainly from two causes: First, The sparsely inhabited condition of the new settlements and the inability of the residents to meet their obligations, except at certain seasons of the year; and Secondly, The promptness and cheerfulness with which the pecuniary relations of physician and patient were recognized and discharged. These causes, happily in the first and unhappily in the second place, no longer exist, and there consequently appears to be no other reason for the continuance of the custom, than that of precedent, a precedent that is in reality rather burdensome than advantageous to the debtor, while to the creditor it is always an inconvenience, and not infrequently involves the loss of an entire debt. It is no idle vaunt, that legitimate medicine is not practiced for money; fortunately, the physician has other motives and other rewards; but still it must not be forgotten, that he and those dependant upon him look to his profession as their livelihood, and his receipts should not be more distant or precarious than those of other persons, particularly as he is expected to pay his own debts as promptly as others, and has no exemption, above his fellows, from pecuniary obligations of every description. It is often stated by those engaged in commercial pursuits, that professional men should not require quick returns for their services, as they have no cash capital at stake. This is an error. Many years usually elapse before the outlay required by three years of pupilage, and the possession of the necessary books and instruments, is once turned. But the absolute amount invested, although necessarily considerable, should weigh little in comparison with the mental anxiety and responsibility that must ever attend the physician; nor does it bear any proportion to the physical hazard involved in exposure to disease and to the elements at any and all hours.

The public are concerned more nearly than they perhaps suppose in the prompt and full compensation of medical men. As has been before stated, legitimate medicine is not practiced for money alone, but the physician wno is harrassed and annoyed by debts he has been obliged to contract, although he may have, upon his books, a large surplus in his favor, is in no frame of mind to give that entire and undivided attention to his patients which their condition may require, and which he would wish to bestow. Another reason for ready payment of the physician exists in the fact, that he renders his services where they are required, not when it will be convenient to him; besides he *gives* largely, not only of his time and skill but also of his means; for the pain and need that meets his eye, often make an appeal to the heart, that suffers no delay. He gives, moreover, to every public charity. There is not a hospital, or an orphan asylum, or any kindred institution, to which his services are not rendered gratuitously and cheerfully. To every appeal for private or public benefit, he is expected to respond, and he does so, largely and liberally in proportion to his means.

Such being the position and such the claims of the medical man, should he not receive a fair proportion of an income, that from the nature of its source is of necessity variable and precarious. This he does not do under the present system of collecting. What is advisable? Shall he claim his fee when he makes his visit, as is done in Great Britain, and in the larger towns on the continent? To this there could be no objection, and with certain exceptions it would be best for all parties—but so radical a change your committee do not wish to urge, save in case of strangers and non-residents. They offer, however, for your serious consideration, the following proposition, viz:

*Resolved*, That it is deemed just and expedient that *all accounts* for medical services, be presented at least as often as every three months.

The financial revulsion of the past year points strongly to the wisdom of such a course. Had this been the usage, many accounts now probably entirely lost, would have been cheerfully and easily settled. A medical bill is always paid more readily, when the remembrance of professional service is fresh in the memory of the recipient. "That short accounts make long friends" is an axiom not less true in medical than in other matters—a bill cheerfully paid is twice paid, while one grudgingly settled, or extracted by force of law, is never discharged satisfactorily to either party. Your committee are fully aware that the resolution they have presented, cannot be made binding, nor would they have it so. They would wish a just discretion to be exercised, but it appears in their judgment, highly desirable that the general usage should be such as advised, and their proposition is, therefore, respectfully committed to the attention of their professional brethren, and to those who are equally interested, the public.

THOS. F. ROCHESTER,
AUSTIN FLINT,
SANDFORD EASTMAN.

On motion, the report was accepted and the committee discharged.

Prof. HAMILTON moved that the sentiments of the report be approved by the meeting, and that the resolution contained therein, be adopted. Seconded and carried.

Prof. WHITE then reported the following case of laceration of the perineum:

Mrs. W., of Niagara county, N. Y., æt. 18 years; primapara. She was confined ten weeks before the operation, with a male child, and delivered, after a difficult labor, with one blade of the forceps. After delivery, the perineum was found to be torn completely through the sphincter ani, dividing also the lower margin of the gut itself. She had no control whatever over fæces or gas.

Her condition before the operation was this: She was exceedingly feeble, and it was necessary to keep the bowels alternately confined with opium and opened by laxatives, in the latter event the contents passing away involuntarily.

On Wednesday, May 10th, she took a dose of castor oil which operated freely.

On May 11th, assisted by Drs. Rochester, Newman and Miner, Dr. White operated in the following manner: The patient was placed in the position for lithotomy, the knees being bent well upon the abdomen, and the parts denuded of hair. She was then brought completely under the influence of chloroform, by Dr. Rochester, and was kept insensible during the operation. The knees being firmly held by Drs. Rochester and Miner, who in turn held and made tense the labia during the incisions, a deep incision was made with the scalpel equal to the length of the fissure, and extending internally at least an inch upon each labium, completely removing the mucous membrane and laying bare the surface. The membrane was then removed from the rectal surface of the fissure, taking care to leave none of it upon any of these surfaces, as a fistula would then be certain to result. The sphincter ani and intervening tissues were then divided obliquely on each side of the centre, extending the incisions an inch or more, until all resistance of the sphincter was overcome. These incisions were made with a blunt pointed bistoury, which was introduced within the anus and guided by the forefinger of the left hand, and thus carried through all the fibres of the muscle, the skin and sub-cutaneous areolar tissue.

The thighs were then approximated, and three quilled sutures introduced by a strong needle extending an inch upwards into the vagina and commencing internally about an inch from the edges of the fissure. These sutures were of twine and doubled, so as to enclose a piece of gum elastic bougie; by means of which, firm pressure was maintained throughout the opposing surfaces. Four interrupted sutures were then introduced superficially, in such a manner as to keep the skin in close contact and secure union by the first intention. The forefingers of each hand were then passed into the vagina and rectum to ascertain whether any fistulous opening remained. Being satisfied that there was none, the parts were sponged and the patient placed in bed in the flexed position upon her left side, and cold water dressings were applied. Perfect quietude was maintained and opium given at intervals of from two to four hours in 1 to 2 gr. doses, in order to constipate the bowels and secure sleep. The strength was sustained by beef essence, quinine, port wine and ale, and the urine drawn off carefully every four or six hours. The following week, on Saturday, the bowels were moved by warm water injections, having remained closed for ten days.

The sutures had been removed on the sixth day, and a careful examination now showed that union was complete. Drs. Rochester, Newman and Miner, also examined and concurred with me as to the success of the operation.

The following week, fourteen days after the operation, the patient left for home, feeling greatly encouraged, and I have since heard from a medical friend, that he considers the cure perfect.

Prof. White remarked that the operation which he had described, was essentially the same as that performed by Isaac Baker Brown. When the fissure is so extensive, much relief is not generally expected from an operation. This view is supported by very high authorities. Churchill does not allude to it, nor does Cooper in his surgical dicticnary. The old operation, as recommended by Dieffenbach, is of doubtful propriety. Dr. W. meant to be understood as speaking of complete division of the perineum, when the fissure is complete, as in the case which he mentioned. Slight lacerations are not important, and soon heal of themselves; a complete laceration, however, through the rectum, is a most loathesome and disgusting disease. Dr. W. has never before been entirely successful in operations upon patients in this condition. The improvements which he has made in the preceding operation are:

*First*, The amount of surface made bare by the knife, which was much greater than usual.

*Second*, The complete division of the sphincter ani, so that the union was not in the least retarded by its contractions. This Dr. W. hesitated to do, being afraid that it would not unite; it did unite, however, thoroughly, and the patient had as perfect control over the contents of the bowels as before the accident. Divide it, and you will escape a great deal of annoyance and injury which would otherwise arise from its contractions.

*Third*, The kind of sutures employed. Formerly the interrupted suture was employed, and occasionally the quilled suture; but before Baker Brown, the stitches were never as deep as in this case, where the parts were kept in most perfect apposition by equable pressure, and the union of the skin was assisted by the few points of the interrupted suture which were superadded.

*Fourth*, The keeping the bowels constipated for such a length of time, which is rather a modern idea. It was formerly sometimes recommended to keep the bowels soluble, and sometimes to keep them closed for two or three days, and then move them. It was never customary to keep them confined as long as ten days.

*Fifth*, Leaving the sutures for a longer time than is common. Dr. White would have been glad to have made trial of the silver wire as recommended by Dr. Sims, of New York, but the case was rather urgent and would not admit of the delay of sending for the wire.

Dr. White also wished to call attention to the treatment likely to prevent laceration of the perineum in child-birth. It has been recommended by a contributor to one of the Southern Journals, to divide the perineum when there was an danger of rupture. This author divided it in the median line, but it has been recommended by Dubois, Challi, and other eminent authorities, to divide it on one side. Dr. W. thought that a proper degree of support, assisted by the relaxing effects of chloroform would almost always be sufficient to make it dilate. He considered chloroform as an exceedingly useful agent in this respect; but should it not dilate, and should it show no disposition to do so, he thought it was the imperative duty of the obstetrician to divide the parts as directed by Dubois, on one or both sides of the median line. A cut of the perineum is a trivial affair, but an extensive laceration is most serious.

Prof. Hamilton wished to express his approval of the operation as made by Dr. White; the same suggestion, as to dividing sphincter, or muscles which would by their contraction, retard union, had been made in operations for fissure of the palate. Next to this in the bold and free paring of the edges, which principle will apply to all like operations. In Dr. Hamilton's opinion, it made no difference what kind of ligature was employed, so long as it was unirritating and not absolutely poisonous, and of sufficient size. It is the smallness of the ligature and the amount of strain put upon it which inflicts the injury. He thought that Dr. Sims had never done himself such injustice as when he gave such an immense superiority to the silver suture, condemning all others.

Prof. Flint wished to inquire in regard to the efficacy of supporting the perineum, and asked in what way rupture could be most certainly avoided. He had thought that the idea of supporting the perineum in every case was rather an antiquated notion.

Prof. White replied, that in a large proportion of cases he believed that it was unnecessary, but in primaparæ much could be done by an upward pressure, preventing the head from impinging too forcibly on the perineum. Challi, De Paul, and others, were right in supposing that the shoulders most frequently lacerated the perineum, after the head had passed through safely. He thought that in a large proportion of cases, too persistent pressure on the perineum was injudicious, but in some cases it had a good influence in making the dilatation more gradual.

Prof. Rochester mentioned a case of labor which he attended when he was a student of medicine. The perineum was exceedingly rigid and unyielding, and Dr. R. supported it continually for about six hours. He was absolutely afraid that the head would be expelled through the rectum, but at last it was safely born, but when the shoulders emerged, the perineum gave way. He had also seen another case of rupture from the passage of the shoulders.

Prof. HAMILTON inquired if a small laceration was not often made by the head, which was enlarged by the shoulders.

Dr. EASTMAN had a case of labor a few weeks since, in which he gave all the support to the perineum which was possible, and in which the perineum was ruptured by the shoulders. He had a case ten days ago where the head was born sometime before the shoulders, and he made careful examination after the head had emerged, and could discover no laceration; but after the birth of the child the perineum was lacerated.

Dr. FLINT, Jr., mentioned a case of injury to the fingers.*

Prof. HAMILTON presented specimens of what he said he should term "warts," or vegetations removed from the vulva of a female. They had been growing about four years, and covered a large part of the labia. Some of them were of great size. Dr. Hamilton had removed them by the knife and scissors, a few weeks before. As to their character or origin he was willing to say more than Ricord dare say of his Paris patients. He knew they were not of venereal origin. These warts Dr. Hamilton had seen growing in various parts of the body; upon the hands; upon the glans penis; about the anus; upon the vulva, &c. He had seen one instance in which they formed at the angles of the mouth in a child. But wherever they grow their character is essentially the same. They are examples of hypertrophy of the papillæ. No doubt, sometimes, there is something more than hypertrophy, the papillæ undergoing a morbid change of structure; they may, even, degenerate into malignancy. The form of these enlargements of the papillæ is sometimes leaf-like, the leaves being set upon the sides of each other like some species of cactus; at other times they are cylindrical, at others, Dr. H. has seen them like a mass of homœopathic pills.

Dr. Hamilton believes all warts, wherever or whenever, occurring, to be essentially the same, yet some are preceded by, or accompanied with venereal diseases, and some are not. How shall we distinguish them? Ricord says that venereal warts are sometimes contagious. This is true, but then the contagion produces not lues, not a chancre, but a wart. A wart existing on a gonorrhœal female, I have known also, to produce a similar wart upon a male. To assert, then, that the venereal wart is contagious, is only to declare what is equally true of all warts. They all may be, and generally are,

* This case is reported in another part of the Journal.

propagated by innoculation. What, then, constitutes the difference between venereal and other warts? Dr. Hamilton repeated that he believed there was no difference in their character, and yet surgeons said the venereal wart had a broader base, and he knew that such was generally the fact. He had at the college, a number of casts which he had himself obtained from different patients, some of which were venereal and some were not. But this fact does not imply of necessity a difference in the wart, so that one may be called "common," and one "specific," as writers have generally done. A boil occurring in a patient suffering under lues, or who has in him the syphilitic taint, will have a broader base; all sores or wounds even, under these circumstances, are slow in maturing, slow in healing, and prone to the deposit of fibrin and to induration. So, also, the base of the wart occurring in a venereal system is prone to the same expansions and indurations.

It will not follow that we can always make the diagnosis, since, occasionally, from various causes, the wart which occurs in a non-venereal system will have a broader base, but such a fact will found to be the exception and not the rule. This one, for example, has a very narrow pedicle.

In cutting them off we ought to remember that they are exceedingly vascular. If smaller they may be removed by a dry powder, and especially by a remedy much used of late, savine and sulphate of copper in powder.

Prof. White remarked that the observations of Dr. Hamilton were new to him, and struck him with a great deal of force. One case which came under his observation, appeared to militate against his views. Two years since he had a patient who was entirely free from the venereal taint, with warty excrescences, which had existed for thirteen years. These had a *broad base.* The husband had never had venereal disease, and had never suffered from warts. Dr. White removed them at 11, A. M. At 1 or 2, there was hæmorrhage, and they tied some vessels, but it could not be arrested. He applied muriated tincture of iron, ice, snow, nitrate of silver, but could not succeed; at length he ceased his applications in despair, and separated the thighs, allowing the cold air access to the parts, when the hæmorrhage ceased. The same persistent oozing occurred to him in an amputation of the penis.

Prof. Hamilton said that the rule which he had mentioned was of course not infallible, that warts might have a broad base and not necessarily be venereal.

Dr. Nichell presented an interesting specimen of a calculus which he had removed from the urethra of a child.

Prof. WHITE reported a case of puerperal convulsions, which he saw at Lancaster. The patient was a primapara, æt. 24; she was delivered June 11th, after a moderately severe labor, of a female child. On the night of the 12th, she was attacked with convulsions, and had nine before noon on the 13th. Her urine was albuminous. She was bled to partial syncope in the semi-erect position, and purged with croton oil. The convulsions increased in severity and frequency, and on the arrival of Dr. White, she had two in the course of twenty-minutes. Pulse 150. Between the convulsions she was entirely comatose with immovable pupils. Dr. W. immediately administered chloroform, with the instantaneous effect of arresting the convulsions and producing sleep. The spasmodic movements ceased, and the pulse began to fall. The administration was continued for five hours, increased at times when she appeared to be restless. At that time the pulse had fallen to 100, and she was able to be aroused for the purpose of taking nourishment. Dr. White then left the patient, directing ¼ gr. of morphine every four hours, and the chloroform to be continued as before, for twelve hours, the patient being kept slightly under its influence, increasing it when she was restless. The urine was also drawn off at intervals of six hours. On the 14th she was much improved; the pulse was 80, and she was calm and quiet. On the 17th the husband called to say that she was convalescent.

Dr. White remarked that the preceding case illustrated the value of chloroform as a remedy in puerperal convulsions. Not that it is to supplant every other mode of treatment, but that it is all-important in connection with them. He did not wish to undervalue phlebotomy in certain cases, or the use of purgatives, especially croton oil; but he wished to insist upon the importance of chloroform. Puerperal convulsions are sui generis, not apoplectic or epileptic. They are almost always associated with albuminuria and dependent upon nervous irritation; this irritation is most effectually counteracted by chloroform; there is no objection to the judicious employment of the other measures which have been mentioned, and no objections to the use of anodynes, but chloroform is always necessary and all-important. Other remedies may be added; anodynes sometimes exert a very beneficial influence in lessening cerebral congestion or irritation.

Prof. ROCHESTER mentioned a case which was relieved by another mode of treatment; this, however, was not so manifestly a case of puerperal convulsions. The patient was in her third pregnancy. Five years ago she was attacked with hemiplegia, which still continued. During the second pregnancy, she was taken with coma and a convulsion in a store, and had a succession of them during the following day. A few days since he was summoned in great haste to see her, in consultation with Dr. Gould, and learned that she had been taken with a sudden pain in the head and had fallen into a comatose condition; the pupils were insensible; pulse 150; great lividity; cold extremities and frothing at the mouth. She could not be aroused from her coma. There was some doubt as to the character of the attack, but on consultation it was determined to bleed, which was done freely, from a large orifice, and in the semi-erect posture. There was immediately a marked change for the better, and the next morning she was able to walk about. She has continued to improve.

Dr. Rochester does not feel absolutely certain as to the character of the seizure; he was disposed to give chloroform to relieve the convulsive twitchings which were present in the case, but was deterred in consideration of the organic disease of the brain which had existed so long.

Prof. White thought that the case was not similar to the one he had described; that there was great doubt as to the precise character of the attack.

Dr. Treat had recently seen a case of convulsive coma in a man who had been working for a length of time in the sun. This man recovered in a few hours by merely putting his head in cold water, and walking him about.

Dr. Wilcox moved that the report of the committee on "the presentation of medical accounts," which was read by Dr. Rochester, be published in some of the daily papers. Seconded and carried.

On motion, the Association then adjourned.

AUSTIN FLINT, Jr., M. D.,
Secretary.

